# Physicians’ Perceptions of the Use of a Chatbot for Information Seeking: Qualitative Study

**DOI:** 10.2196/15185

**Published:** 2020-11-10

**Authors:** Jason Koman, Khristina Fauvelle, Stéphane Schuck, Nathalie Texier, Adel Mebarki

**Affiliations:** 1 Kap Code Paris France; 2 CNRS PASSAGES Bordeaux France; 3 Bordeaux Population Health Research Center University of Bordeaux Inserm Bordeaux France; 4 Sanofi Aventis Gentilly Cedex France

**Keywords:** health, digital health, innovation, conversational agent, decision support system, qualitative research, chatbot, bot, medical drugs, prescription, risk minimization measures

## Abstract

**Background:**

Seeking medical information can be an issue for physicians. In the specific context of medical practice, chatbots are hypothesized to present additional value for providing information quickly, particularly as far as drug risk minimization measures are concerned.

**Objective:**

This qualitative study aimed to elicit physicians’ perceptions of a pilot version of a chatbot used in the context of drug information and risk minimization measures.

**Methods:**

General practitioners and specialists were recruited across France to participate in individual semistructured interviews. Interviews were recorded, transcribed, and analyzed using a horizontal thematic analysis approach.

**Results:**

Eight general practitioners and 2 specialists participated. The tone and ergonomics of the pilot version were appreciated by physicians. However, all participants emphasized the importance of getting exhaustive, trustworthy answers when interacting with a chatbot.

**Conclusions:**

The chatbot was perceived as a useful and innovative tool that could easily be integrated into routine medical practice and could help health professionals when seeking information on drug and risk minimization measures.

## Introduction

A conversational agent, also known as a “chatbot,” is an artificial system that can converse with a human user through automated message exchange [1]. In order to interact in a natural way with the user, it employs a question and answer database [2]. Commonly used in marketing as a consumers’ guide, chatbots also have been developed in the health field in several applications aimed mostly towards patients [3-11]. However, little scientific research has examined the use of conversational agents through physicians’ viewpoints [12-15], and none have taken into account medical information addressed to health professionals. It has been shown that nearly all physicians use the internet for medical information seeking [16,17] but have to cope with an increasing flow of medical information [18]. Too much information to scan could be a barrier to respond to a defined medical question [16,17]; thus, seeking health care information can be an issue for physicians since their information sources can be fragmented, incomplete, and not easy to find [19]. Credibility of the source, relevance, unlimited access, speed, and ease of use are the main criteria for information seeking among physicians [17]. A national survey conducted in France in 2017 showed that the prescription and delivery methods of drugs could be incorrectly followed by health professionals, despite the risk minimization measures campaign conducted at a national level [20]. Providing medical information to health professionals through a conversational agent could help to detect pharmacovigilance cases early and to reinforce the proper intake of medication by patients. More specifically, chatbots could be deployed as a complementary solution to provide information about drugs with the purpose of minimizing drug risks. In France, drug risk minimization measures are generally recommended by the European Pharmacovigilance Risk Assessment Committee and adapted by the French National Agency for Medicines and Health Products Safety (Agence Nationale de Sécurité du Médicament et des Produits de Santé) to regulate medical practice and ensure safe, efficient intake of drugs [21].

To improve drug information access and awareness of drug risk minimization measures among physicians, a pilot version of a chatbot was developed and tested. The chatbot was expected to improve drug information access and awareness of drug risk minimization measures. This qualitative study aimed to improve the chatbot to meet physicians’ needs and expectations. User participation [22] was used to elicit physicians’ perceptions of the chatbot through the use of the pilot version. Gathering more in-depth qualitative information on these topics may be useful to help develop a conversational agent that meets physicians’ needs and expectations.

## Methods

### Study Design

The study focused on the pilot version of a text-based chatbot developed by an international pharmaceutical company. This was qualitative research that employed individual, in-depth, semistructured interviews to explore physicians’ opinions and perceptions of a chatbot. The aim of the chatbot was to provide quick, 24-hour access to information on drugs for physicians to improve their medical practice and the application of recommendations regarding drug risk minimization measures among patients. Thus, all information provided by the chatbot was sourced from official regulatory documents issued by French health authorities to ensure patient security. The chatbot was accessible online through a web browser. When logged in, it displayed a dialog box on the right side of the screen and a female figure in the central area. Answers provided by the chatbot followed pre-established topics related to drugs (eg, treatment initiation, discontinuing treatment, treatment renewal, documentation or information that can be downloaded and obtained by the user). When asked a question, the chatbot provided a direct answer based on its database. When a question was not sufficiently focused, the chatbot proposed between 2 and 4 categories related to the question. The user then had to choose between those categories by typing the corresponding number. When the chatbot was asked a question that was not available in its database or not understood because of a typing error, the user was alerted. In this latter case, the user had to reformulate the question or ask a new question related to pre-established topics. As a pilot version, the chatbot initially focused on one drug. Further information can be added and delivered when the final version is deployed; the chatbot is meant to be used by physicians who seek quick access to information in the presence of a patient or when preparing a consultation.

General practitioners were approached initially as the conversational agent was developed for this specific population. Specialists were also approached because some of their patients used drugs subject to risk minimization measures.

### Recruitment and Participants

Participants were recruited on a voluntary basis after being asked by email whether they were interested in “taking part in a project that will test an innovative digital health tool.” Their email addresses were available from a database of health practitioners who had already participated in previous digital epidemiological studies. The interviewer had not met the participants before the interview. General practitioners were recruited to represent various situations such as urban and rural general practice. Specialists were recruited from either public or private practice. Thematic saturation was not taken into account since the study aimed to improve the chatbot through physicians’ needs and expectations in an explorative way without being representative.

### Data Collection

#### Interview Design

Semistructured interviews were conducted during June 2018 and July 2018 and took place primarily in participants’ medical offices. At the beginning of the interview, each participant received a brief summary of the study from the interviewer, and sociodemographic data (gender, age, profession, and workplace) were collected. Interviews were scheduled for approximatively 1 hour.

To standardize the interviews, they were divided into 3 sections: introduction, introduction of the chatbot, and conclusion.

#### Introduction

The introduction included knowledge about the use of drugs and recommendations about risk minimization measures and acceptability of a chatbot within their daily practice.

#### Presentation of the Chatbot

The chatbot was presented through the example of 3 preselected input phrases based on various situations: “How to accompany a pregnant woman taking this medicine?”, “I want to renew the prescription of a patient.”, “Does this drug decrease the impact of contraception?” Test sessions were conducted as follows. After the introduction, the interviewer logged in to the chatbot with a confidential password. In the dialog box, introductory text was displayed and read aloud by the interviewer to explain the purpose of the chatbot. Then, preselected input phrases were first inputted by the interviewer to show how the chatbot worked. Participants were then asked to try the chatbot by searching for information on drugs as they would do during a real consultation, using their own terminology. Questions were collected by the interviewer after a physician concluded the test, but participants were left free to use the conversational agent as much as they wanted during the rest of the interview. Since it was a pilot version, participants did not have access to the conversational agent before or after the interview. Moreover, the test was different from a real-case scenario since participants were not totally autonomous in using the chatbot and had to share their experience with the interviewer.

#### Conclusion

During the conclusion, feedback was collected on the user experience with the chatbot. Physicians were asked about the relevance and appreciation of the tested tool as well as their needs for and expectations of the chatbot.

#### Template

The interviewer followed a semistructured interview template composed of the following themes: (1) knowledge about drug risk minimization measures and acceptability of digital health within their daily practice (Section 1), (2) relevance and appreciation of the chatbot to facilitate information acquisition (Section 3), and (3) needs for and expectations of the tested chatbot (Section 3).

### Data Analysis

Interviews were recorded using a digital audio recorder after receiving participant agreement. They were fully transcribed and anonymized by the interviewer. Transcripts were organized, sorted, and coded using a systematic thematic analysis approach [23]. Main themes were developed and identified by the authors as patterns emerged within data [24]. Then, they were graphically represented through the use of Visual Understanding Environment (VUE) software. For each participant, data were summarized in one table following the main themes identified during the interviews. Quotations that were not related to the chatbot were excluded from analysis. Data from each theme were gathered in another table to proceed with the horizontal thematic analysis by highlighting sentences expressed by participants on each topic.

## Results

### Demographics

A total of 10 health professionals participated in individual, in-depth, semistructured interviews in France.

The sample size included 8 general practitioners and 2 specialists. The average age of the participants was 51.5 years. There were 7 men and 3 women; 6 participants worked in an urban area only, 3 worked in a rural area only, and 1 worked in both urban and rural areas. All participants were following patients who needed routine drug prescriptions including risk minimization measures.

All participants reported being vigilant about new recommendations for prescription including risk minimization measures before using the chatbot. Seven physicians reported that they sought information on drugs subject to risk minimization measures only when their patients were directly concerned, when they were confronted with a particular case, or when adverse events had been reported to them:

I have access to information obviously, but I get more interested when I am confronted with the problem.

Man, in practice for 35 years

They considered the information on drug risk minimization measures easy to find but fragmented. The main source of information they reported was the internet, followed by information from health authorities (letters, email), databases on drugs, medical journals, and communications from laboratories (press, visits). Exchanges with other practitioners had also been cited as sources of information:

The easiest and most accessible information is the summary of characteristic products, but it is in rather indigestible to read since it’s really big.

Man, in practice for 11 years

Generally, I do research spontaneously. I’m looking out on the internet when I have a question about a treatment or when I’m not sure about a question a patient asked me, we can look together.

Woman, in practice for 8 years

I get information from National Agency for Medicines and Health Products Safety, from health authorities, and from laboratories as well. I receive either emails or letters.

Man, in practice for 30 years

### Central Themes

#### Emerged Topics

Several themes and subthemes emerged from the interviews. The central themes were (1) achievement by man including ergonomics and format of asking; (2) achievement by tool including design, tone, and form of presentation; (3) content of output including the amount of information, clarity, and accuracy; and (4) user needs. These topics are discussed with illustrative quotes in the following paragraphs.

#### Achievement by Man

In total, 52 questions were asked to the chatbot by participants. Of the 52 questions formulated by the physicians (Multimedia Appendix 1) during tests, 24 were answered by the pilot version of the conversational agent. One-third of the 24 answers (7/24) were obtained on the first try, while the other two-thirds (17/24) needed a complementary question. Furthermore, 28 questions were not understood by the conversational agent, and 8 questions were not included in the conversational agent themes. Questions addressed to the conversational agent were about prescription, medical treatment renewal, drug side effects, drug interactions, and records on drug products.

Some physicians judged the ergonomics favorably due to simplicity and ease of use:

This is correct. The style is sober, quiet; it is not aggressive. It is simple and readable.

Man, in practice for 36 years

Yes, it is really easy to use. The ergonomics are really good.

Man, in practice for 11 years

Over half (6/10) of the participants found that the chatbot lacked intuitiveness and fluidity to access information:

The answer is clear, but I don’t know, there is something with the fluidity... If I compare with where I’m usually looking for information, there is one section for each topic, and I get instant access. Here, the answers are too robotic.

Woman, in practice for 24 years

I would say this is not intuitive. Everyone knows how to use a computer. But the ergonomics... I don’t know, there is just a login page, then the home screen. If it is just that, there is no ergonomics. It would be nice to have a website plan.

Male, in practice for 30 years

The size of the dialog box was also cited as improvable:

There is a part of the screen that is not used; the dialog box looks really small compared to the rest. It could occupy a larger space, which would allow a bigger typeface.

Man, in practice for 11 years

The window to type our questions is too small. If you enter a long sentence, you can’t see the beginning of your sentence, which is bothering. Indeed, the window is too small and the typeface as well.

Man, in practice for 36 years

#### Achievement by Tool

Most of the participants reported that the colors and icon of the chatbot were too simple, but the global design was judged serious and professional, which was appreciated:

You could give us the possibility to customize the design by choosing between 3 or 4 different colors. Maybe it should be a bit more cheerful. It looks seriously made, but a little too dark.

Man, 18 years of practice

I like the font; it is quite nice. It looks as if everything was made to be relatively neutral. This is good because the tool can’t be visually aggressive during a consultation. This is not really attractive; it is quite neutral, but this is adapted because visual elements catching your eyes all the time can be tiring. This is better.

Man, in practice for 11 years

Seven participants reported that the tone of the answers was positive. However, the display of the answers provided by the conversational agent needed to be more concise according to half (5/10) of the physicians:

It speaks from a medical point of view so there is no problem with the tone.

Man, in practice for 36 years

It needs a display with bold characters so we can reach key points by reading through it diagonally

Man, in practice for 23 years

#### Content of Output

The response delay from the chatbot was acceptable for 9 of 10 participants, but 5 physicians were not satisfied with the length of the information, which was too broad to read or to access:

It is a little bit long because every time it asked me for a clarification, it seems that it doesn’t understand.

Woman, specialist in practice for 24 years

This is accurate but it is too much talking. Two lines should be enough; this is too long in my opinion.

Woman, in practice for 24 years

Even though all participants found the information provided by the chatbot very clear, the main issue pointed out by physicians was the accuracy of the answers. Obtained answers did not provide a sufficient amount of information. Thus, even if they trusted the provided information, it was considered as too generic and not relevant enough:

We need clear and concise scientific information. We need to know globally what to do; then, we translate the information but at least it needs to be precise. Because for now, it is a little bit general; this is too elementary.

Man, in practice for 35 years

The answers are understandable; it comes out quickly; it comes out clear. The only inconvenience is that it is not the answer I was looking for.

Man, in practice for 18 years

It is centered on the developers’ insight, not from a physician’s insight. This is almost a tool for a patient actually. Physicians have much more questions than the chatbot can answer for now.

Man, in practice for 11 years

#### User Needs

Participants identified pros and cons of the chatbot after they tested it (Table 1). Pros were the concept, ergonomics, assistance for diagnosis, and time savings. Cons were the natural language comprehension issues, data security, and the threat to health care professions.

**Table 1:** Pros and cons of the chatbot.

Observation and examples		Example excerpts
**Pros**		
	Relevant concept: give fast and precise information	“In my opinion, the concept is really interesting. Why? Because this is provisioning a tool in which we can get precise and fast information. This is a concept that seems very interesting and very relevant to me.” [Man, in practice for 35 years]
	Ergonomic tool: can be utilized as a research tool	“The ergonomics, the conciseness…Yes, the shape. You call this a conversational agent; to me, it is a good research engine.” [Man, in practice for 11 years]
	Diagnosis helping: can provide assistance to medical care	“The fact that it can give answers when we have a question is interesting. That doesn’t mean we’ll necessarily follow the answer but at least it gives a direction. Tools that can help medical care or to diagnose, I find great.” [Woman, in practice for 24 years]
	Time-saving	“There are only benefits anyway. This is instantaneous, verified information. It saves us time. A consultation is time-consuming; we need to get immediate answers. It has already happened to me that I had to call a laboratory to get an answer, and I was told that someone would call me back. Here, I get my answer almost directly” [Man, in practice for 30 years]
**Cons**		
	Natural language comprehension issues: can be difficult to access the right information	“The tree structure to get to the information. That is to say… If I get some information, in what degree is it intuitive? Does it need to be questioned in a precise way, or can I ask my questions without thinking about the formulation? Can it answer my everyday needs?” [Man, in practice for 18 years]
	Medical data issue: can be dangerous for medical data security	“Of course, there is hacking. I can’t take control of a doctor, but I can get control of a conversational agent without problems. All the security part, as powerful it can be, can always be breached if someone intends to do it. That’s the limit.” [Man, in practice for 23 years]
	Threatens health care professionals: a chatbot could replace doctors	“Let’s say that, in 10 years, we are told that our job will be replaced; it is a nightmare. We studied for 10 years, not 2 years, but 10 years. If we are told that we will be replaced by artificial intelligence, it does not make anyone dream.” [Man, in practice for 23 years]

Nine participants estimated that this tool would be easily integrated in their routine practice when fully developed, especially if it could provide fast, trustful, easily accessible information on a range of medical topics:

It is simple; it is practical. When it has acquired more vocabulary, I think it will be an efficient tool.

Woman, in practice for 8 years

This is a tool in which we can get precise and fast information. This is a concept that seems very interesting and very relevant to me”

Man, in practice for 35 years

However, half (5/10) of the participants considered that the chatbot did not meet their needs. The main reason was the natural language comprehension issues:

It needs to be accurate and well-edocumented because, during a consultation and in our daily routine, we don’t have much time. Not having enough time means we need to get to the point; so, we need fast, specific information”

Man, in practice for 35 years

If I get information, what is the degree of intuitiveness? The machine has to adapt, because when I’m doing my job with a patient in front of me, I can’t be focused on how I should formulate my question to the chatbot.

Man, in practice for 18 years

During a consultation, everything has to be done quickly, I need information quickly. If the chatbot doesn’t understand me, I put it aside to get the information elsewhere.

Woman, in practice for 24 years

Participants reported areas to improve the chatbot prototype (Textbox 1). Suggestions were made about the content of the conversational agent, its display and appearance, and the extensions of use that could be made. Suggestions included highlighting the most important information provided by the chatbot, the possibility to provide an information sheet to the patients, or integrating medical themes beyond drug risk minimization measures.

**Suggestions made by general practitioners and specialists for improvement of the conversational agent.**

Display and appearance

- Highlight important information in the answers
- Possibility to choose interface colors

Content

- Provide [printed] information to the patients
- Regular updates

Extensions of use

- Ask questions orally
- Integrate more themes (eg, drug interactions, pathologies)
- Declare side effects

## Discussion

### Principal Findings

The study was conducted to elicit physicians’ perceptions of a chatbot that is meant to improve drug information access and awareness of drug risk minimization measures. A qualitative approach was chosen to collect detailed data on how the chatbot was used and perceived by physicians on a small scale and needs and expectations for the chatbot. As a pilot version, the chatbot did not meet physicians’ expectations.

Overall, the findings demonstrated that physicians’ needs towards information delivered by the chatbot were the reliability of sources, precise information, and speed of access. This is consistent with previous research demonstrating that all these features are critical to physicians when seeking information [17]. Physicians were particularly vigilant to the conversational agent content. Even though they did appreciate getting information on risk minimization measures and drugs, they had difficulties accessing the right information because of natural language understanding issues. As a tool based on artificial intelligence, physicians also expected that the chatbot would understand natural language. Previous research demonstrated that over half of physicians believed that chatbots lack the intelligence or knowledge to accurately assess the user [13], which can be an issue regarding speed and ease of use when using the chatbot.

The chatbot format was also appreciated, as it could easily be integrated into physicians’ routine practice, either during or before a consultation with a patient. This was judged as innovative since most chatbots in health care are developed to provide information for patients [3-11]. Access to information is also considered easier with a conversational agent than with a classic drug reference database. First, it was considered to provide reliable information in a practical way. Second, it made information accessible so that physicians could quickly find what they were looking for and, consequently, save time. In that way, using a chatbot can improve medical care from physicians’ perspectives. Shared decision making may increase the effectiveness of a treatment if the patient is given a sufficient and appropriate amount of information [25]. In this regard, the chatbot could help medical care.

It was pointed out that the conversational agent did not always respond to the questions they formulated, either because it did not recognize the everyday language employed by the users or because the answers, while based on official regulatory documents, were too broad without highlighting the most important parts of information. However, they were aware that the chatbot was still in an experimental stage. Regarding this fact, most practitioners were willing to use the conversational agent in its fully developed version if this tool remains easy to use, secure, and easily accessible.

Areas of improvement for the conversational agent proposed by medical practitioners included a better understanding of the questions formulated, highlighting the most important information, and better ergonomics.

### Limitations

This market research is not without limitations. As an exploratory study with a sample size of 10 medical practitioners, findings are not generalizable to the entire population of medical practitioners. This was a qualitative study conducted with semistructured interviews, which allowed us to explore tendencies and opinions on the usage of a conversational agent. The average age of our sample (51.5 years) was slightly above the average age of the medical practitioner population in France, which was 50.8 years in 2018 [19]. The majority of our sample was male practitioners (7/10, 70%), which does not reflect the distribution between male (53%) and female (47%) medical practitioners in France in 2018 [19].

In addition, medical practitioners who were recruited had already participated in other digital epidemiological studies carried out by the same research team, and it is possible that they were more receptive to new health technology. In other words, while our sampling method was not meant to be representative, this specific market research may be prone to researcher bias.

Finally, because of the confidentiality policy, the drug associated with the chatbot could not be cited in the paper. However, we believe that it did not interfere with the results, which were meant to elicit physicians’ perceptions of the chatbot.

### Conclusions

According to the results of this study, it appears that chatbots could be a solution for quick and easily accessible information. By reinforcing the knowledge on drugs based on official and institutional recommendations, a chatbot could be used to enhance compliance with the drug risk minimization measure within the physician population. In particular, this chatbot prototype was perceived by medical practitioners as a useful, acceptable, innovative tool that could easily be integrated in their daily medical practice. Finally, even though the chatbot prototype could not be used as it was because of insufficient information in the database, findings suggest that physicians are willing to use a chatbot not only for prescription but also to get information on drug interactions or to obtain assistance within medical care for complex pathology or disease management. A future challenge for the chatbot should be to accommodate physicians’ needs for accurate, concise information based on official regulatory documents that ensure patients’ security.

## Appendix

Multimedia Appendix 1 Questions tested by physicians.

## References

[1] Amato F, Marrone S, Moscato V, Piantadosi G, Picariello A, Sansone C (2017). Chatbots Meet eHealth: Automatizing Healthcare. http://ceur-ws.org/Vol-1982/paper6.pdf.

[2] Corti K, Gillespie A (2015). A truly human interface: interacting face-to-face with someone whose words are determined by a computer program. Front Psychol.

[3] Gaffney H, Mansell W, Tai S (2019). Conversational Agents in the Treatment of Mental Health Problems: Mixed-Method Systematic Review. JMIR Ment Health.

[4] D'Alfonso S, Santesteban-Echarri O, Rice S, Wadley G, Lederman R, Miles C, Gleeson J, Alvarez-Jimenez M (2017). Artificial Intelligence-Assisted Online Social Therapy for Youth Mental Health. Front Psychol.

[5] Lokman A, Zain J, Komputer F, Perisian K (2009). Designing a Chatbot for Diabetic Patients.

[6] Crutzen R, Peters GY, Portugal SD, Fisser EM, Grolleman JJ (2011). An artificially intelligent chat agent that answers adolescents' questions related to sex, drugs, and alcohol: an exploratory study. J Adolesc Health.

[7] Comendador BEV, Francisco BMB, Medenilla JS, Nacion SMT, Serac TBE (2015). Pharmabot: A Pediatric Generic Medicine Consultant Chatbot. JOACE.

[8] Ireland D, Atay C, Liddle J, Bradford D, Lee H, Rushin O, Mullins T, Angus D, Wiles J, McBride S, Vogel A (2016). Hello Harlie: Enabling Speech Monitoring Through Chat-Bot Conversations. Stud Health Technol Inform.

[9] Stieger M, Nißen M, Rüegger D, Kowatsch T, Flückiger C, Allemand M (2018). PEACH, a smartphone- and conversational agent-based coaching intervention for intentional personality change: study protocol of a randomized, wait-list controlled trial. BMC Psychol.

[10] Ghosh S, Bhatia S, Bhatia A (2018). Quro: Facilitating User Symptom Check Using a Personalised Chatbot-Oriented Dialogue System. Stud Health Technol Inform.

[11] Hess GI, Fricker G, Denecke K (2019). Improving and Evaluating eMMA's Communication Skills: A Chatbot for Managing Medication. Stud Health Technol Inform.

[12] Cameron G, Cameron D, Megaw G, Bond R, Mulvenna M, O'Neill S, Armour C, McTear M (2018). Best practices for designing chatbots in mental healthcare? A case study on iHelpr.

[13] Palanica A, Flaschner P, Thommandram A, Li M, Fossat Y (2019). Physicians' Perceptions of Chatbots in Health Care: Cross-Sectional Web-Based Survey. J Med Internet Res.

[14] Feldman MJ, Hoffer EP, Barnett GO, Kim RJ, Famiglietti KT, Chueh HC (2012). Impact of a computer-based diagnostic decision support tool on the differential diagnoses of medicine residents. J Grad Med Educ.

[15] Laranjo L, Dunn AG, Tong HL, Kocaballi AB, Chen J, Bashir R, Surian D, Gallego B, Magrabi F, Lau AYS, Coiera E (2018). Conversational agents in healthcare: a systematic review. J Am Med Inform Assoc.

[16] Casebeer L, Bennett N, Kristofco R, Carillo A, Centor R (2002). Physician Internet medical information seeking and on-line continuing education use patterns. J Contin Educ Health Prof.

[17] Bennett NL, Casebeer LL, Kristofco RE, Strasser SM (2004). Physicians' Internet information-seeking behaviors. J Contin Educ Health Prof.

[18] Nylenna M, Aasland O (2000). Primary care physicians and their information-seeking behaviour. Scand J Prim Health Care.

[19] Begaud B, Costagiola D (2013). Rapport sur la surveillance et la promotion du bon usage du medicament en France. French Ministry of Solidarity and Health.

[20] Sanofi Aventis France (2017). Evaluation auprès des pharmaciens d'officine des mesures de minimisation du risqué consistant en des nouvelles conditions de prescription et de délivrance (CPD) du valproate. Synthesis from National Agency for Medicines and Health Products Safety.

[21] (2017). Mesures additionnelles de minimisation des risques. National Agency for Medicines and Health Products Safety.

[22] Barki H, Hartwick J (1994). Measuring User Participation, User Involvement, and User Attitude. MIS Quarterly.

[23] Guest G, MacQueen KM, Namey EE (2011). Introduction. Applied Thematic Analysis.

[24] Richards L (2019). Readme First For A User's Guide To Qualitative Methods.

[25] Coulter A, Entwistle V, Gilbert D (1999). Sharing decisions with patients: is the information good enough?. BMJ.

