# Supplementary material for: Physicians’ Perceptions of the Use of a Chatbot for Information Seeking: Qualitative Study
Source: J Med Internet Res. 2020 Nov 10;22(11):e15185. doi: 10.2196/15185 (PMC7685916; doi:10.2196/15185)
Supplement: Multimedia Appendix 1 [file jmir_v22i11e15185_app1.docx]

*Table 1. Questions tested by physicians.*

| Interview | *Question*  *number* | *Question asked* | *Chatbot provided an answer* | *Clarification needed* |
| --- | --- | --- | --- | --- |
| Interview 1 | *1* | *« Prescription among adolescent women? »* | *No* | *Not understood by chatbot* |
|  | *2* | *« Renewing a prescription for an adolescent »* | *No* | *Not understood by chatbot* |
|  | *3* | *« Does the drug decreases contraception efficiency?”* | *Yes* | *Yes* |
| Interview 2 | *4* | *« Side effect for pregnant women »* | *Yes* | *Yes* |
|  | *5* | *« Drug risk »* | *No* | *Not available* |
|  | *6* | *« Medical surveillance »* | *No* | *Not available* |
|  | *7* | *« Drug side effects »* | *No* | *Not available* |
|  | *8* | *« Drug potential risks »* | *Yes* | *No* |
|  | *9* | *« Treatment benefits »* | *No* | *Not understood by chatbot* |
|  | *10* | *« Drug and contraception »* | *Yes* | *Yes* |
|  | *11* | *« Drug potential risks »* | *Yes* | *Yes* |
|  | *12* | *« Drug side effects »* | *No* | *Not understood by chatbot* |
|  | *13* | *« Treatment and blood test »* | *No* | *Not understood by chatbot* |
|  | *14* | *« Drug characteristics »* | *No* | *Not understood by chatbot* |
| Interview 3 | *15* | *« In which case can’t I prescribe the drug? »* | *No* | *Not understood by chatbot* |
|  | *16* | *« Drug and contraception »* | *Yes* | *Yes* |
|  | *17* | *« Drug under contraception »* | *Yes* | *Yes* |
|  | *18* | *« Prescription recommendations »* | *Yes* | *Yes* |
| Interview 4 | *19* | *« Drug posology »* | *Yes* | *No* |
|  | *20* | *« Drug and vaccination »* | *Yes* | *Yes* |
|  | *21* | *« Drug interactions »* | *Yes* | *Yes* |
|  | *22* | *« How to accompany a patient? »* | *Yes* | *No* |
|  | *23* | *« I want to renew the prescription for a patient »* | *Yes* | *No* |
|  | *24* | *« Does the drug decrease contraception efficiency? »* | *Yes* | *Yes* |
| Interview 5 | *25* | *« Can I prescribe the generic drugs? »* | *Yes* | *Yes* |
|  | *26* | *« Generic »* | *No* | *Not understood by chatbot* |
|  | *27* | *« Patient file »* | *No* | *Not understood by chatbot* |
|  | *28* | *« Drug proper use »* | *No* | *Not understood by chatbot* |
|  | *29* | *« Capsule color »* | *No* | *Not understood by chatbot* |
|  | *30* | *« Drug picture »* | *No* | *Not understood by chatbot* |
|  | *31* | *« Drug dispenser »* | *No* | *Not understood by chatbot* |
|  | *32* | *« Drug interaction with antibiotics »* | *No* | *Not understood by chatbot* |
|  | *33* | *« Drug interaction with antimalarial treatment »* | *No* | *Not available* |
|  | *34* | *« Interactions »* | *Yes* | *Yes* |
| Interview 6 | *35* | *« Drug interactions »* | *Yes* | *Yes* |
|  | *36* | *« Drug risks »* | *Yes* | *No* |
|  | *37* | *« Drug side effects »* | *No* | *Not understood by chatbot* |
|  | *38* | *« When should I interrupt the treatment? »* | *No* | *Not understood by chatbot* |
| Interview 7 | *39* | *« How to switch the treatment? »* | *Yes* | *No* |
|  | *40* | *« Can I prescribe to an elderly person »* | *Yes* | *Yes* |
|  | *41* | *« Prescription condition for an elderly person »* | *No* | *Not understood by chatbot* |
| Interview 8 | *42* | *« How to stop the treatment? »* | *Yes* | *No* |
|  | *43* | *« Drug and pregnancy »* | *Yes* | *Yes* |
|  | *44* | *« Pregnancy and side effects »* | *No* | *Not available* |
| Interview 9 | *45* | *« Can I modify drug posology »* | *No* | *Not understood by chatbot* |
|  | *46* | *« Modify drug posology »* | *No* | *Not available* |
|  | *47* | *« Renewal »* | *Yes* | *Yes* |
|  | *48* | *« Can I modify the posology »* | *No* | *Not available* |
|  | *49* | *« Consultation frequency with a specialist »* | *No* | *Not available* |
| Interview 10 | *50* | *« What are the renewal rules for the pharmacist? »* | *Yes* | *No* |
|  | *51* | *« Treatment renewal »* | *Yes* | *No* |
|  | *52* | *« Pharmacy and renewal »* | *No* | *Not understood by chatbot* |
